# Improving the Efficacy of Magnetic Nanoparticle-Mediated Hyperthermia Using Trapezoidal Pulsed Electromagnetic Fields as an In Vitro Anticancer Treatment in Melanoma and Glioblastoma Multiforme Cell Lines

**DOI:** 10.3390/ijms242115933

**Published:** 2023-11-03

**Authors:** Lilia Souiade, Javier Domingo-Diez, Cesar Alcaide, Berta Gámez, Linarejos Gámez, Milagros Ramos, José Javier Serrano Olmedo

**Affiliations:** 1 Center for Biomedical Technology (CTB), Universidad Politécnica de Madrid (UPM), 28040 Madrid, Spain; lilia.souiade@ctb.upm.es (L.S.); javier.domingo@ctb.upm.es (J.D.-D.); cesar.alcaide@ctb.upm.es (C.A.); milagros.ramos@ctb.upm.es (M.R.); 2 Escula Técnica Superior de Ingenieros Industriales, Universidad Politécnica de Madrid, 28006 Madrid, Spain; berta.gamez@upm.es (B.G.); linarejos.gamez@upm.es (L.G.); 3 Centro de Investigación Biomédica en Red para Bioingeniería, Biomateriales y Nanomedicina, Instituto de Salud Carlos III, 28029 Madrid, Spain

**Keywords:** hyperthermia, magnetic nanoparticles, nonharmonic signals, alternating magnetic field, cancer, melanoma, glioblastoma, nanomedicine

## Abstract

Magnetic hyperthermia (MHT) is an oncological therapy that uses magnetic nanoparticles (MNPs) to generate localized heat under a low-frequency alternating magnetic field (AMF). Recently, trapezoidal pulsed alternating magnetic fields (TPAMFs) have proven their efficacy in enhancing the efficiency of heating in MHT as compared to the sinusoidal one. Our study aims to compare the TPAMF waveform’s killing effect against the sinusoidal waveform in B16F10 and CT2A cell lines to determine more efficient waveforms in causing cell death. For that purpose, we used MNPs and different AMF waveforms: trapezoidal (TP), almost-square (TS), triangular (TR), and sinusoidal signal (SN). MNPs at 1 and 4 mg/mL did not affect cell viability during treatment. The exposition of B16F10 and CT2A cells to only AMF showed nonsignificant mortality. Hence, the synergetic effect of the AMF and MNPs causes the observed cell death. Among the explored cases, the nonharmonic signals demonstrated better efficacy than the SN one as an MHT treatment. This study has revealed that the application of TP, TS, or TR waveforms is more efficient and has considerable capability to increase cancer cell death compared to the traditional sinusoidal treatment. Overall, we can conclude that the application of nonharmonic signals enhances MHT treatment efficiency against tumor cells.

## 1. Introduction

**Figure 1:**
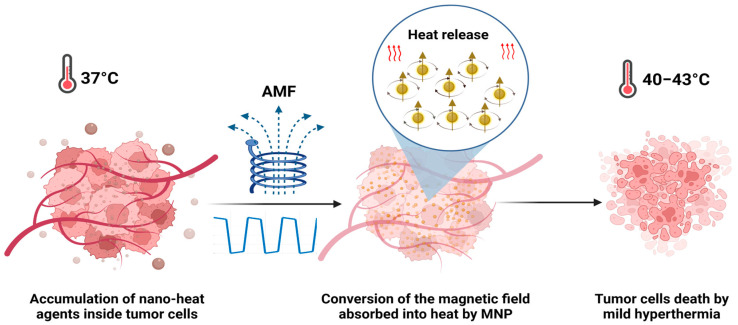
MNPs internalized into the tumor are remotely activated by an AMF to reach high temperatures (MHT) and eliminate the tumor cells.

Progress in cancer treatment remains a high priority, considering that cancer is still the leading cause of death all over the world. In 2020, there were nearly 19.3 million new cancer cases and 10.0 million deaths per year. In 2040, it is expected to be 28.4 million cases [1]. Recent years have seen intense applications and device development based on electromagnetic fields (EMFs). At the biomedical level, the use of EMFs has known significant investigation in many aspects, involving disease detection, diagnosis, cancer therapy [2], and the noninvasive treatment of infections, such as prosthetic joint infections [3]. Many studies have been directed towards hyperthermia treatment against cancer using magnetic fields, radiofrequency, and microwaves [4,5]. While EMFs have potential therapeutic applications, it is important to understand the potential risks associated with their exposure at high intensity and to use them only under appropriate conditions and with appropriate safeguards. Alternating magnetic fields (AMFs) can have in vitro and in vivo effects depending on a variety of factors (frequency, intensity, and duration of exposure). These effects can be both beneficial and harmful, and they can vary depending on the biological system being exposed. One of the most well-known biological effects of AMFs is the induction of eddy currents inside the tissues. At higher frequencies and intensities, they can generate heat in tissues [6]. Magnetic hyperthermia (MHT) is based on the application of an AMF at low frequencies (<1 MHz), which is considered to be safe [4,5,6,7]. MHT has been clinically tested and proven effective as a treatment for recurring glioblastoma multiforme, combined with radiotherapy [8] for prostate cancer [9] and against melanoma, in combination with chemotherapy [10]. MHT has several advantages over traditional cancer therapies; it is less harmful to healthy cells due to the local heat release in the tumoral area after the administration of magnetic nanoparticles (MNPs). MHT can be used in combination with other treatments, such as immunotherapy [11], chemotherapy, or radiation therapy, to enhance their efficacy [12,13]. Additionally, it is known that tumor cells are more sensitive to heating than healthy cells and that the heat produced in hyperthermia by exposing MNPs to an AMF can induce cell death or alter the growth and differentiation of these tumor cells (Figure 1). Tumor cells subjected to a mild hyperthermia temperature range (40–43 °C) die by apoptosis, while the thermoablation temperature range (>45 °C) leads to necrosis [14,15].

The most widely used MNPs for MHT are iron oxide nanoparticles (magnetite Fe_3_O_4_ and maghemite γ-Fe_2_O_3_) of nanoscopic size so that they magnetically behave as a superparamagnetic material for what are called SPIONs; superparamagnetic iron oxide nanoparticles (SPIONs). In MHT, the MNPs can be remotely activated by an AMF due to their intrinsic ability to convert magnetic energy into heat. Two dissipative mechanisms define the MNPs’ heating behavior: the Néel relaxation and the Brownian relaxation. SPION heating is described by the specific absorption rate (SAR) [16]. The heating efficiency of these nanoheat agents depends strongly on their magnetic properties, which are influenced by the concentration, viscosity, and aggregation [17]. The main interest of these MNPs are their biocompatibility, safety, and also their degradation following natural paths [18,19]. For their interesting effects and extensive uses, they entered the medical market, and currently, there is an approved cancer nanomedicine product called NanoTherm^®^ for hyperthermic treatment against glioblastoma [20]. Another liposomal nanomedicine product was developed for hyperthermia but is not yet approved, which is called ThermoDox^®^, for advanced hepatocellular carcinoma treatment [21]. MNPs can be employed as well for controlled drug release, as MRI contrast agents, for cell separation and cellular labeling [22], for photothermal therapy [23], and magneto-mechanical destruction therapy [24].

During the hyperthermal treatment, the heating efficiency can be improved by carefully considering the adjustment of MNP properties and EMF radiation parameters, which will absolutely increase the damage to the cancer. Previously reported preclinical hyperthermic studies have shown promising results and significant damage to cancer cells after treatments where conventional sinusoidal waveforms with different magnetic field strengths and frequencies have been used. These studies have been carried out on several cell lines and animal models and also with different types of MNPs [14]. The idea of enhancing heating efficiency by optimizing the signal slope has recently emerged. A numerical study has shown that the use of modified driving field waveforms provides impressive outcomes on the SAR from an assembly of iron oxide nanoparticle Neel relaxations and that the TP and TS waveforms are promising [25]. The heating efficiency enhancement in MHT has been experimentally explored using nonharmonic waveforms to prove that their application to excite the nanoheat agents optimizes the heating power performance, where TP and TS signals showed a higher heat generation performance compared to the SN signal [26]. The experimental results showed superior heat generation by TS that achieved over 71% of the thermal power generation efficiency compared to SN and 45% by TP. Therefore, the optimization of hyperthermia by applying nonsinusoidal signals beside the enhancement of the NP features could allow it to overcome the issues posed in both preclinical and clinical studies and permit NPs to reach their optimal alignment to optimize the killing effect on tumor cells and the MNP internalization process. The present work was carried out to achieve actionable results to create a new way to fight cancer and enhance MHT-based anticancer treatments. For this purpose, research was conducted on two cancer cell lines, murine B16F10 melanoma and CT2A malignant glioma, subjected to different waveforms (nonharmonic waveforms against the sinusoidal waveforms) generated by an enhanced AMF generator.

## 2. Results

### 2.1. In Vitro Cytotoxicity of APS-SPION

**Figure 2:**
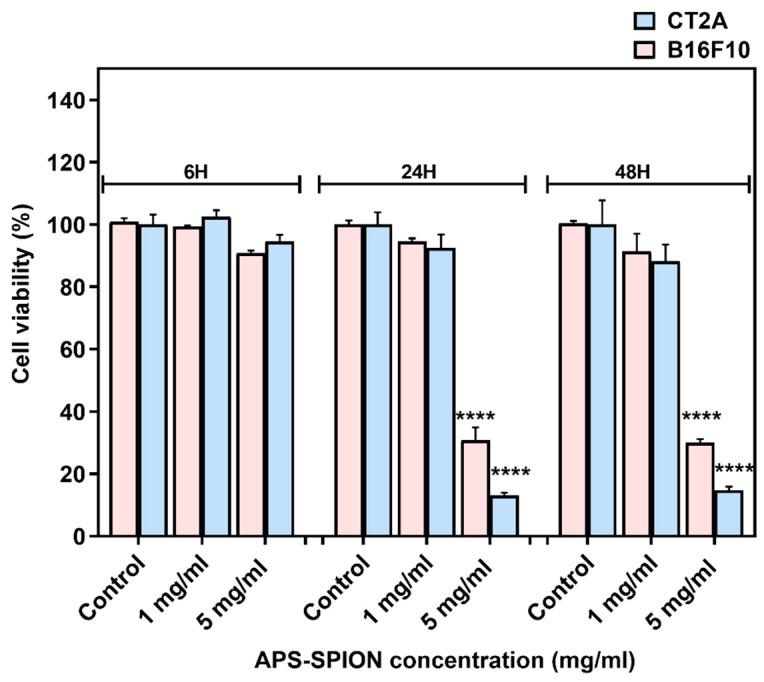
B16F10 and CT2A cell viability evaluated by XTT assay after incubation with APS-SPION at 1 and 5 mg/mL for 6 h, 24 h, and 48 h. Data represent the mean ± SEM of three independent experiments (n = 3). ANOVA, post hoc Fisher’s LSD test; **** *p* < 0.0001.

The viability of B16F10 and CT2A cells incubated with APS-SPION at different concentrations (1 and 5 mg/mL) on B16F10 and CT2A cells are depicted in Figure 2. Both cell lines exhibited similar responses to the presence of MNPs, although CT2A cells showed lower cell viability when incubated with MNPs at 5 mg/mL for 24 h and 48 h compared to B16F10 cells. XTT results showed a nonsignificant decrease in cell viability when both cell lines were incubated with MNPs at 1 mg/mL during all time periods tested. However, when cells were incubated with the APS-SPION at 5 mg/mL, a significant reduction in cell viability was observed after 24 h and 48 h of incubation in both cell lines, whilst cell viability remained over 90% within 6 h.

### 2.2. Safety of the AMF Applicator

**Figure 3:**
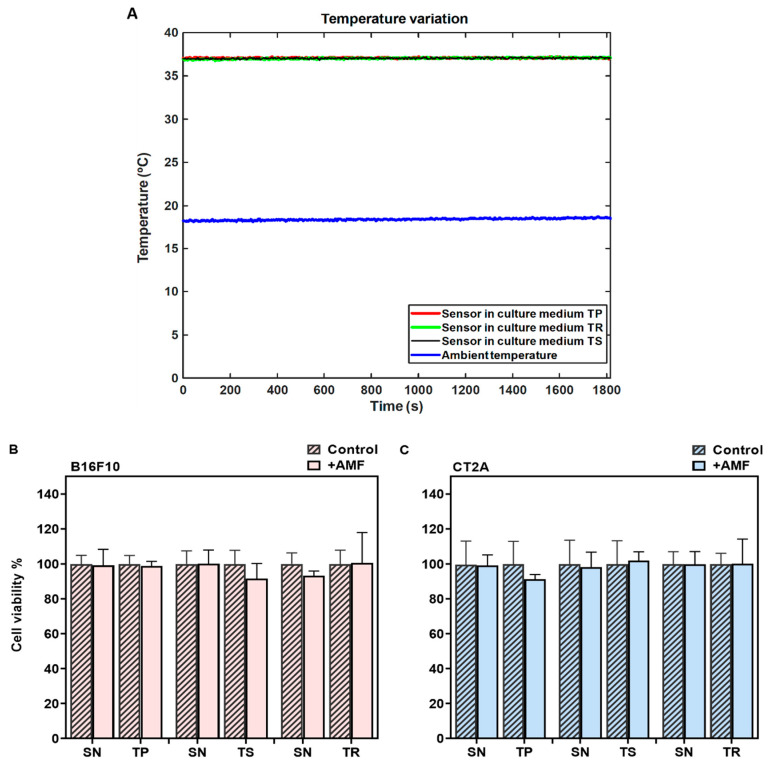
Determination of the safety of the MHT applicator using different signals. (**A**) Data of temperature measurement during hyperthermia treatment without APS-SPION. The red, black, and green curves represent the cell culture medium temperature for different nonharmonic signals. The blue curve represents the room temperature. (**B**) B16F10 and (**C**) CT2A cell viability evaluated with Calcein/PI 24 h after different waveform AMF application for 30 min (+AMF) compared to cells not exposed to AMF (control). AMF waveforms SN: sinusoidal, TP: trapezoidal, TS: almost square, TR: triangular.

In MHT treatments, the cell samples must be thermally isolated, especially from the solenoid coil that shows harsh heating during the EMF exposure. In order to demonstrate that the cell damage during MHT treatment is only due to the power generated by the APS-SPION exposed to an AMF, a PLA sample holder connected to the water pump was designed to keep the temperature of the cell cultures stable at 37 °C during AMF exposure. Therefore, cells were cultured in petri dishes of 35 mm and placed inside the holder. The temperature of the culture dishes was measured throughout AMF exposure using an optical temperature probe immersed in the medium. A second optical probe was placed within a beaker of water at room temperature. Figure 3A represents the temperature variation during the exposure to the TP signal (2.78 mT/300 kHz), TS signal (2.14 mT/300 kHz), and TR signal (3.42 mT/300 kHz) for 30 min. The temperature of the sample remains stable at around 37 °C during the AMF exposure, as shown in Figure 3A, proving to be adequate thermal isolation. Hence, the sample is isolated from the superficial thermal interferences. The viability of the cells exposed to AMF in the absence of MNPs was assessed using a Calcein-AM/PI staining assay and compared to their corresponding controls (cells without AMF exposure). The results from the Calcein-AM/PI assay (Figure 3B,C) show that the exposure to AMF did not affect cell viability in any of the signals analyzed.

### 2.3. Cell Uptake of MNPs

**Figure 4:**
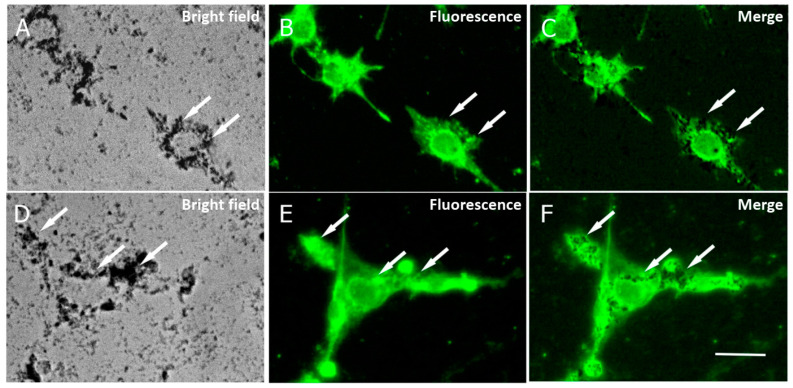
Cellular localization of MNPs in B16F10 (**A**–**C**) and CT2A (**D**–**F**) cells. Cells were incubated with MNPs for 24 h and then stained with an anti-CD63 to mark lysosomes. Photomicrographs show MNPs in bright field microscopy as black dots (**A**,**D**) and lysosomes by fluorescence microscopy, marked in green (**B**,**E**). Note that cells with strong lysosome labeling show large accumulations of MNPs (arrows). Panels (**C**,**F**) show the merged images from panels (**A**,**B**) and (**D**,**E**), respectively. Scale bar: 20 µm.

The internalization of APS-SPION into cancer cells has been described previously across different cellular types [27,28], indicating that macropinocytosis is the primary mechanism by which the nanoparticles are internalized inside cells and that the APS coating does not induce any endosome/lysosome damage [19]. To assess the uptake of the nanoparticles by B16F10 and CT2A cells in our study, cells were preincubated with 10.6 nm APS-SPION for 24 h and evaluated by microscopy. APS-SPION accumulations were detected under bright field microscopy (Figure 4). Nanoparticles were observed outside and also inside the cells as black cytoplasmic spots of distinct sizes (arrows in Figure 4A,D). Figure 4 shows the intracellular distribution of APS-SPION visualized in cells with strong labeling for lysosomes (Figure 4B,E), as determined by immunostaining with the anti-CD63 green fluorescent antibody that specifically stains the lysosomes. The resulting image confirmed the presence of APS-SPION within the cancer cells. Our results from the internalization of MNPs in Figure 4 further support the previous research on cellular uptake of these nanoparticles directly inside cells under a visible light microscope [29] or by confocal microscopy, ICP-OES, TEM, and Prussian blue staining [27,28]. An evident intracellular uptake of MNPs was observed by the images extracted from Calcein/PI staining consistent with other studies carried out in different cell lines [29].

### 2.4. Evaluation of the In Vitro Magnetic Hyperthermia Treatment

**Figure 5:**
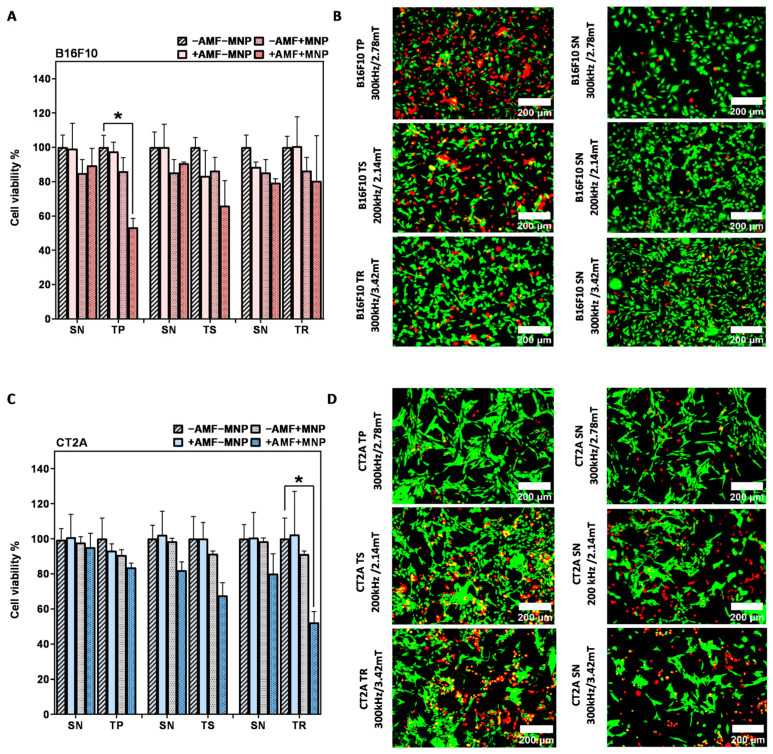
After magnetic hyperthermia cell viability evaluation in B16F10 and CT2A cell lines. Cell viability counting results obtained using fluorescence microscopy with B16F10 (**A**) and CT2A (**C**) preincubated with 1 mg/mL of APS-SPION and exposed for 30 min to different AMF signals (SN/TP: 300 kHz and 2.78 mT, SN/TR: 300 kHz and 3.42 mT, SN/TS: 200 kHz and 2.14 mT). Cells were stained with Calcein-AM and PI 24 h after MHT treatments. Live and dead cells were quantified by Image J software version 1.8.0 for each experimental condition. Representative images obtained by fluorescence microscopy of the treated group (+AMF+MNP) cells stained with Calcein (green) and PI (red) (**B**,**D**). Scale bar: 200 μm. ANOVA, post hoc Fisher’s LSD test; * *p* < 0.05.

Cell viability results 24 h after MHT treatment of B16F10 and CT2A cells preincubated with MNPs at 1 mg/mL and exposed to the AMF for 30 min are depicted in Figure 5A,C, respectively. The graphs show the cell viability with all the predefined experimental conditions (−AMF−MNP, −AMF+MNP, +AMF−MNP, +AMF+MNP). The cytotoxicity of APS-SPION on both cell lines evaluated by Calcein/PI was slightly different from those obtained with the XTT results assay (Figure 2), but, still, MNPs were found to be not cytotoxic, with cell viability rates of more than 80% in B16F10 cells (Figure 5A) and more than 90% in CT2A cells (Figure 5C). This slight difference may be due to the different tests used. The cell viability of cells exposed to only AMF remains, as well, higher than 80% with B16F10 and 90% with CT2A (Figure 5A and Figure 5C, respectively). These observations prove that the cell viability reduction observed after MHT treatments is produced by the effect of the combination of AMF and SPION. The cell viability after MHT treatment was clearly different between the treatment using nonsinusoidal signal (TP, TS, and TR) and sinusoidal signal (SN), where the treatments using TP, TR, and TS are shown to be dominant and to exert more efficient effect to damage the cancer cells (Figure 5).

The application of AMF using nonsinusoidal waveforms (TP, TS, and TR) produced in both cell lines a greater decrease in cell viability compared to the application of SN waveform (Figure 5). The TP-AMF exposure of B16F10 loaded with 1 mg/mL APS-SPION produced a significant decrease in cell viability (53 ± 5%) compared to the application of SN-AMF that reduced cell viability to 89 ± 9% at 300 kHz/2.78 mT. TP was the most effective waveform in B16F10 cells (Figure 5A). Cell viability was nonsignificantly reduced with the application of a TS-AMF (65 ± 14%) and SN-AMF (90 ± 0.8) at 200 kHz/2.14 mT, whereas the reduction in cell numbers was nearly equal by the application of TR-AMF and SN-AMF waveforms (over 80 ± 14%) at 300 kHz/3.42 mT. In CT2A cells, the AMF application to cells loaded with 1 mg/mL APS-SPION produced a minimal decrease in cell viability by both TP-AMF (83 ± 2%) over SN-AMF (95 ± 8%) at 300 kHz/2.78 mT and by TS-AMF (67 ± 7%) over SN-AMF (82 ± 4%) at 200 kHz/2.14 mT. The TR-AMF waveform was more effective on CT2A at 1 mg/mL and led to a significant cell viability reduction of 52 ± 6% over the SN-AMF (80 ± 11%) at 300 kHz/3.42 mT (Figure 5C). The images of the treated groups +AMF + MNP with both cancer cell lines obtained by fluorescent microscope are presented in Figure 5B,D to further corroborate the damage effects of the hyperthermic treatment in B16F10 and CT2A cells by the nonsinusoidal signals. In these images, live cells are shown in green and dead cells in red after Calcein-AM and PI staining, respectively.

**Figure 6:**
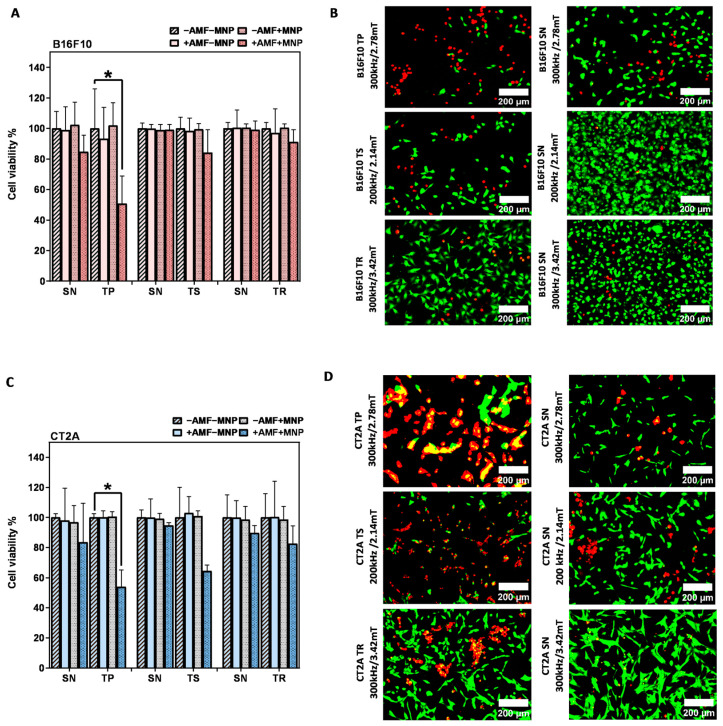
After magnetic hyperthermia cell viability evaluation in B16F10 and CT2A cell lines. Cell viability counting results obtained using fluorescence microscopy with B16F10 (**A**) and CT2A (**C**) preincubated with 4 mg/mL of APS-SPION and exposed for 30 min to different AMF signals (SN/TP: 300 kHz and 2.78 mT, SN/TR: 300 kHz and 3.42 mT, SN/TS: 200 kHz and 2.14 mT). Cells were stained with Calcein-AM and PI directly after MHT treatments. Live and dead cells were quantified using Image J software version 1.8.0 for each experimental condition. Representative images, obtained using fluorescence microscopy, of the treated group (+AMF+MNP) cells stained with Calcein (green) and PI (red) (**B**,**D**). Scale bar: 200 μm. ANOVA, post hoc Fisher’s LSD test; * *p* < 0.05.

To further evaluate the effects of MHT treatment with the nonsinusoidal signals on the cell damage, the same experiment was carried out in B16F10 and CT2A with higher concentrations of MNPs, and the cell viability was directly evaluated after the hyperthermic treatment (0 h). The concentration was increased to 4 mg/mL, and the incubation time was reduced to 2 h to prevent any cytotoxic effect, according to previous cytotoxicity results obtained with APS-SPION (Figure 2). This hyperthermia treatment strategy was adopted in order to maximize the treatment quality in vitro and to achieve an effective cellular uptake quickly. The results of Figure 6 show that the cell viability reduction generated by the application of nonsinusoidal signals was obviously superior with all waveforms (TP, TS, and TR) compared to the SN waveform. In contrast to Figure 5A in B16F10 cells, the TR signal denoted the same mortality as the SN signal. The cumulative heat effect generated in cells by applying the MHT treatment planning at 4 mg/mL for 30 min of exposure to AMF was more efficient than by applying it at 1 mg/mL in most of the experimental cases.

Cell viability results after MHT treatment applied on B16F10 and CT2A cells preincubated with 4 mg/mL MNPs and exposed for 30 min to AMFs using different waveforms are shown in Figure 6. The graphs show the cell viability with all the predefined experimental conditions (−AMF−MNP, −AMF+MNP, +AMF−MNP, +AMF+MNP). No cytotoxicity was exerted by APS-SPION at 4 mg/mL, and cell viability was kept at around 96–100% for both cell lines. The exposure of cells to only AMF was shown to be safe as well, and the viability remained around 93–100%. Hence, a decrease in cellular viability greater than 7% is the result of the coaction between APS-SPION and AMF signals during MHT treatment. In Figure 6A,C, the TP-AMF exposition of 4 mg/mL MNP-loaded B16F10 cells and CT2A cells induced a significant decrease in cell viability by 50 ± 18% and 53 ± 11% at 300 kHz/2.78 mT, respectively. Cell viability was nonsignificantly reduced by TS-AMF and TR-AMF over SN-AMF at 200 kHz/2.14 mT and at 300 kHz/3.42 mT, respectively, in both cancer cell lines. Again, the images of the treated group +AMF+MNP with B16F10 and CT2A cell lines obtained by fluorescent microscope are presented in Figure 6B,D to further indicate the damage effects of the anticancer hyperthermic treatment by the nonsinusoidal signals, where live cells show as green and dead cells as red with Calcein-AM and PI staining.

**Figure 7:**
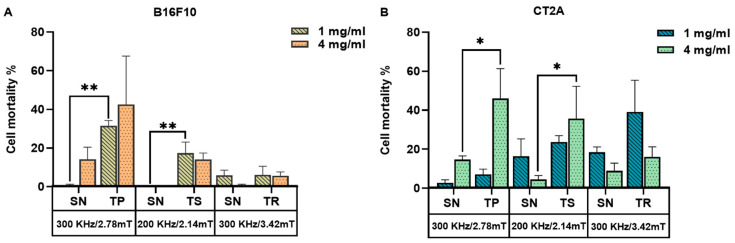
Cell death rates of B16F10 (**A**) and CT2A (**B**) cells preincubated with MNPs at 1 mg/mL and 4 mg/mL and exposed for 30 min to AMFs using different waveforms (TP/SN, TS/SN, TR/SN) at the indicated parameters. To evaluate cell mortality, cells were stained with Calcein-AM/PI, and fluorescence microscopy images were quantified using Image J software. Data represent the mean ± SEM of three independent experiments (n = 3). ANOVA, post hoc Fisher’s LSD test; * *p* < 0.05, ** *p* < 0.01.

To recapitulate, Figure 7 represents the mortality produced by the coaction of SPION and different AMF signals, which are nonsinusoidal (TP, TS, TR) and sinusoidal (SN) applied at the same frequency and amplitude on B16F10 and CT2A preincubated with MNPs 1 mg/mL and 4 mg/mL. Cell mortality was evaluated 24 h after MHT treatment when cells were incubated with MNPs at 1 mg/mL (Figure 7A) and directly after treatment when MNPs were incubated at 4 mg/mL (Figure 7B). The cell death achieved by TP, TS, and TR AMF application had a superior impact among all the cases tested compared with the cell death produced by the corresponding SN waveform. TP and TS waveforms significantly reduced cell viability in B16F10 cells preincubated with MNPs at 1 mg/mL (Figure 7A), except for TR-AMF MHT treatment 24 h later on B16F10 at 1 mg/mL, which gave nearly equal rate of mortality with SN-AMF at 300 kHz/3.42 mT. Comparatively, the cell mortality decrease observed directly after TP-AMF and TS-AMF exposure on CT2A preincubated at 4 mg/mL was highly significant over SN-AMF as opposed to cell viability decrease 24 h after MHT treatment in the same cells preincubated with MNPs at 1 mg/mL. In summary, promising results were achieved by the applicability of the nonsinusoidal signals (TP, TS, and TR) at the best conditions (4 mg/mL over 2 h of incubation with cells), where cell mortality was directly evaluated after exposure to AMF. These outcomes seem to be due to the following reasons. Firstly, the cells have recovered and proliferated 24 h after the treatment since the rate of proliferation is unlimited in the presence of nutrients as they are tumoral cells. In addition, the decrease in pH due to cell proliferation, serum in culture media, and incubation time were considered as factors causing MNP aggregation [30]. The MNP aggregation decreases nanoparticle-induced MHT [31]. During hyperthermia, the heat shock proteins are also activated in response to MHT, which plays a role in fighting the effects of increased temperature by preventing protein denaturation, promoting cellular survival, and overcoming cell death [32,33]. Finally, it seems that the signals of lower slopes (TR) at low MNP concentration require superior frequencies to increase the efficiency of treatment, which confirms the assumption by [34]. In fact, certain requirements must be met when using the magnetic field generator; a suitable power supply circuit is required to produce nonsinusoidal waveforms at high amplitude and frequency, preventing interferences.

However, the nonsinusoidal signals have revealed their strong effect and superiority over the sinusoidal signal for the same frequency and amplitude. The direct evaluation of MHT outcome against cancer during in vitro tests seems to be more accurate and makes it possible to prevent interferences that may appear in the long term of the experiment. Furthermore, there are some differences between MHT effects on B16F10 and CT2A, which is expected since it has been described previously that the cancer cells exert different responses to thermal stress [35] and that the cell death pathway may depend on the location of magnetic nanoparticles and the intracellular concentration inside cells [36].

## 3. Discussion

Our study demonstrates an enhancement of the efficiency of MHT for killing cancer cells with nonharmonic signals. The TP, TS, and TR are more effective and capable of enhancing the efficiency of MHT against tumoral cells; these results support the previously published studies by [25,26,37]. This is consistent with another experimental study that has revealed the capability of the nonsinusoidal waveforms to enhance the energy dissipated by MNPs excited by a nonsinusoidal signal as compared to the energy dissipated by MNPs excited by a conventional signal [26]. The application of these new waveforms was found to increase heat production efficiency to above 71% by the TS and 45% by the TP over the SN signal, while the TR signal trailed behind in increasing the heat efficiency and was less powerful than SN. The adequate mathematical model that describes the heat generation by MNPs when subjected to nonsinusoidal waveforms and its relationship with inducing higher cell mortality is still unknown. But we assume that by increasing the slope of the waveform, the rates of magnetic field energy absorption and conversion to heat by MNPs increase considerably due to a faster reorientation and alignment of nanoparticles’ magnetic moments with the direction of the magnetic field, compared to the sinusoidal, which leads to an increase in cell death. The outcomes of our study of the SN waveform have shown mild or no cell damage due to the use of lower AMF intensity. In addition, the temperature of the cell samples in the presence of MNP remained stable at around 37 °C while we obtained more degrees of temperature with the nonsinusoidal waveforms (1–1.5 °C). Therefore, the treatment with the SN waveform did not induce considerable cell damage. The intensity was not an obstacle for us since we aimed to improve the treatment efficacy by applying nonsinusoidal waveforms of a high slope, which is an alternative to increasing the heating efficiency and, hence, the efficacy of the treatment.

For the in vitro tests, nonsinusoidal waveforms’ applicability in MHT was found to increase cell death significantly on the B16F10 cell line to 31 ± 2% by TP-AMF and to 17 ± 5% by TS-AMF at 1 mg/mL and 30 min of exposition to AMF. In addition, the cell death was significantly increased to 46 ± 15% in CT2A by TP-AMF for 30 min of exposition at 4 mg/mL. The TP and TS both have a strong effect on CT2A at 4 mg/mL and 30 min of exposure to AMF.

The applicability of the conventional signals during MHT treatment is often not effective. SK-Hep1 hepatocellular carcinoma cells were treated with MHT at 5 mg/mL for 24 h using an SN-AMF signal at 43.5 kHz/14.35 mT for 30 min exposure time, and nonsignificant mortality was seen after treatment (20% of cell death) [38]. Neither an increase in temperature of the culture medium nor a significant reduction in cell viability was observed following the exposure of murine pancreatic adenocarcinoma Pan02 loaded with APS-MNP (0.25 mg/mL) to the AMF at 250 kHz/31.41 mT [31]. In our results, cell death produced with SN-AMF was not significant, while MHT treatments using TP, TS, and TR signals reached a highly superior mortality. Among the explored cases, MHT using nonsinusoidal waveforms is particularly promising for anticancer therapy owing to the enhancement of antitumor killing effects. In addition, the TP or TS could be more efficient waveforms due to the increased slope. Hyperthermia may affect cell viability by even tiny changes in temperature. Less than a half-degree increase in temperature can negatively impact cell viability [39]. The application of an AC field with a strength of 30.5 kA/m and 233 kHz has reduced cell viability without any perceptible rise in temperature [40]. Since MNPs are internalized in closed lysosomes distributed in the cytoplasm at different sizes, as depicted in Figure 4C,F, and no cytotoxicity was observed with MNP or AMF separately, the cell death found in our study and produced by TP, TS, and TR signals is generated by the synergistic combination of APS-MNPs and AMF.

It has been previously described that very local intracellular heat release by MNPs induces protein denaturation, DNA damage, and depolymerization of cytoskeletal components [41]. During the treatment by MHT, local temperature increments on the surface of MNPs at thermosensitive intracellular sites will obviously lead to cell death. The nucleolar and ribosomal DNA is one of the possible highly thermosensitive sites [42]. A maximum temperature of 8 °C was measured at 100 kHz/30 mT for an exposition time of 12 min on the surface of the nanoheaters inside cells without any appreciable extracellular temperature. The maximum determined intracellular heat generation in the vicinity of MNPs was lower (5.9 °C) than what was measured on the surface of MNPs (8.0 °C) using a nanothermometer and required double time to reach it [43]. This local heat generation is sufficient to produce a significant cell death using effective nonsinusoidal signals (TP, TS) over the conventional signal, which is considerably improved when the frequency and amplitude are raised to the highest levels tolerated for human use. However, temperature assessment around the surface of MNPs or a single MNP in the intracellular and extracellular media remains a challenge, and accurate measurement tools are needed. Our obtained data further support the concept that local MHT therapy with temperature hot-spot generation at the surface of the nanoparticles is responsible for the induction of cellular mortality without any temperature change in the media [43,44,45,46]. In vitro studies have demonstrated that heat is not the only agent responsible for provoking cell death following MHT treatment and that some contributing mechanisms are related to an increase in lysosomal membrane permeability by targeted MNPs under an AMF, which matches up with an augmentation of reactive oxygen species (ROS) production and an increase in the cytosolic activity of the lysosomal protease cathepsin D and causes cell death [47,48]. Lysosomes contain hydrolytic enzymes, and any perturbation of the lysosomal membrane leads to the release of the lysosomal enzymes into the cytoplasm of the cell, which includes proteolytic enzymes of the cathepsin family. This, in turn, activates several intracellular cascades that promote the lysosome-dependent cell death (LDCD) [49]. It has been found that in parallel to the decrease in cell viability, there is an altered expression of procaspases, production of reactive oxygen species, and altered mRNA expression of Ki-67, TOP2A, and TPX2 after MHT exposure [35]. Therefore, the MHT mechanism of action is thought to be due to the fact that the external electromagnetic energy is converted to not only heat but to mechanical effects that cause the vibration of MNP inside cells and organelles membrane, which provoke the membranes to lose their integrity, as demonstrated in [50]. Moreover, it has been discussed that AMF exposure enhances the MNPs’ internalization inside cells; thus, the nonsinusoidal (TP, TS, TR) signals may contribute to better improving this internalization compared to the conventional signals. It is a new challenge to study the effects of the new signals (TP, TS, TR) on cells and how they may better cross cell membranes, which could have a role in increasing the sensitivity of cancer cells to hyperthermic treatment [36]. All the mentioned mechanisms contribute by the end to decrease in one way or another the cell viability. Our results have demonstrated the capability of the nonharmonic signals to reach greater efficiency of the therapeutic mechanism by producing larger cellular stress effects.

## 4. Materials and Methods

### 4.1. Experimental Device

**Figure 8:**
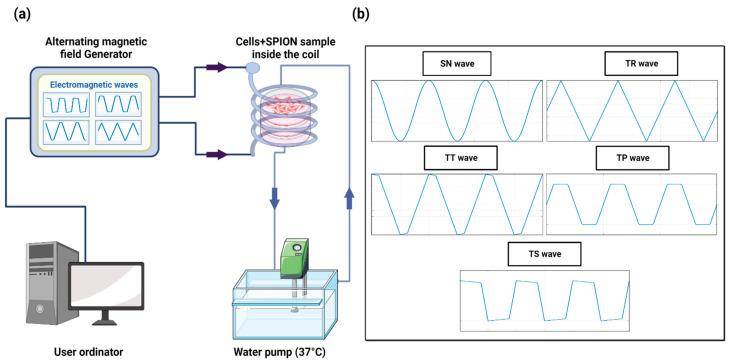
(**a**) Schematic design of the hyperthermia device based on a high-frequency AMF system. (**b**) Harmonic and nonharmonic waveforms generated by the AMF device.

The MHT device was developed in our laboratory [51] and is capable of generating nonharmonic waveforms, specifically these types: Trapezoidal-Square-TS (25% of shift phase), Trapezoidal-TP (50% of shift phase), Trapezoidal-Triangular-TT (75% of shift phase), and Triangular-TR (0% of shift phase), with frequencies ranging from 100 kHz to 1 MHz, plus the SN signal. The AMF is generated through a solenoid copper coil with an inner diameter of 6 cm and outer diameter of 7 cm, which surrounds a closed circulating water system made by PLA and heated up to 37 °C using a water pump to maintain the stable conditions of the cell samples. The temperature was measured using a flexible fiber-optic thermometer (m3300 Biomedical Lab Kit, LumaSense Technologies, Santa Clara, CA, USA). The experimental setup is shown in Figure 8.

### 4.2. Magnetic Nanoparticles

**Figure 9:**
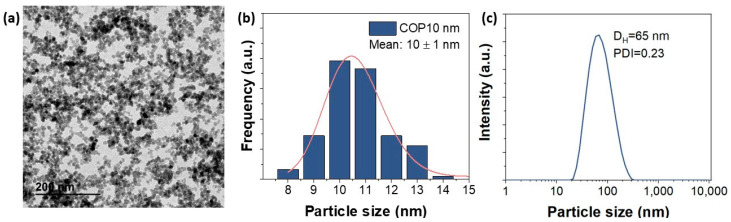
(**a**) Transmission electron microscopy (TEM) micrographs of APS-SPIONs. (**b**) Size distribution of APS-SPIONs in water with TEM and (**c**) Hydrodynamic size at pH 7 measured using dynamic light scattering (DLS).

In our study, we worked with positively charged spherical SPIONs of 10.6 nm (standard deviation = 0.2) donated by Maria del Puerto Morales (Figure 9). At this small size, the nanoparticles will achieve a better performance for MHT application at low field amplitude (<5 mT) and also exhibit excellent colloidal stability in liquid medium [22]. The synthesis method of APS-SPIONs has been previously mentioned in [29], which follows Massart’s coprecipitation protocol [52] for synthesizing maghemite nanoparticles. In brief, the preparation of 10 nm nanoparticles requires the addition of a mixture of 445 mL containing FeCl_3·_6H_2_O (0.09 moles) and FeCl_2_·4H_2_O (0.054 moles) to 75 mL of alkaline medium (NH4OH 25%) under vigorous stirring and at room temperature. Next, using magnetic decantation, the precipitate was washed three times with distilled water. After that, a protocol of oxidation and activation of the surface of the particles was followed by an acidic treatment using 300 mL of HNO_3_ (2 M) with stirring (15 min), then the supernatant was removed using magnetic separation and pouring in 75 mL of Fe (NO_3_)_3_ (1 M) and 130 mL of water, heating to boiling temperature (30 min) and cooling to room temperature and removing the supernatant again. Finally, 300 mL of HNO_3_ (2 M) was added, and the mixture was washed several times with water. To modify the surface of the particles and obtain the APS coating (positively charged), 1.22 mL of APS was slowly added (10 µL/s) to a 20 mL mixture of particles (28 g Fe_2_O_3_/L) in methanol under vigorous stirring for 12 h. Methanol was removed with a rotary evaporator. The hydrodynamic size and the evolution of the zeta potential versus the pH were evaluated in a ZETASIZER NANO-ZS device (Malvern Panalytical, Cambridge, UK). The hydrodynamic size was measured at pH 7 and at room temperature from a dilute suspension of the sample in a standard cuvette.

### 4.3. Cancer Cell Line

The murine B16 melanoma model is the most used for preclinical studies, and it is extracted from a C57BL/6J mouse by repeating the injection process of mice with B16 (malign astrocytoma) 10 times. B16F10 is an extremely aggressive tumor, and it tends to first metastasize to the lungs. It has been shown that in cell culture, the B16F10 presents a heterogeneous population, of which the size varies depending on the stage of maturation. In addition, the melanoma cells can be present in three different sizes: 9, 12, and 15 µm. More abundant are the more mature cells, with a bigger size [53]. They are transformed cell lines (unlimited cell divisions) that begin to secrete melanin after a transformation that occurs when cells grow up to 100% confluence, turning the cell culture medium to a black color.

The mouse CT2A glioblastoma cell line was obtained from a malignant astrocytoma. The tumor was achieved through the implantation of a highly carcinogenic hydrocarbon (methylcholanthrene) inside the brains of C57/BL6 mice [54]. In vitro, the cells grow adherent to the substrate as a monolayer, an essential key for their proliferation. These cells are characterized by an elongated shape compared to B16F10 and die at high confluence.

### 4.4. Cell Culture

The B16F10 and the CT2A cancer cell lines were cultivated in DMEM (Gibco, Billings, MT, USA) supplemented with 10% heat-inactivated fetal bovine serum (FBS), 2 mM of Glutamine (Gibco, USA), 100 units/mL antibiotics of penicillin and 100 µg/mL of streptomycin, and nonessential amino acids. The cell lines were incubated in a humidified atmosphere with 5% CO_2_ at 37 °C until achieving a density around 85 to 95% of confluence.

### 4.5. Experimental Protocol of In Vitro Magnetic Hyperthermia

**Figure 10:**
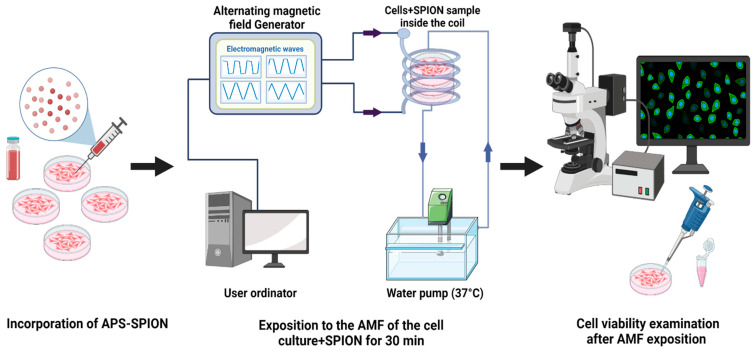
Experimental protocol to apply MHT.

To assess the MHT effect by exciting nanoparticles under an AMF, B16F10 and CT2A cells were grown on 35 mm culture dishes P35. Briefly, the protocol (Figure 10) involves seeding of 1.6 × 10^4^ cells/cm^2^ and 3 × 10^4^ cells/cm^2^ for B16F10 and CT2A, respectively. The cell culture dishes were divided into four groups: cells without any treatment (−AMF−MNP), cells exposed to AMF without nanoparticles (+AMF−MNP), cells incubated with nanoparticles (−AMF+MNP), and cells incubated with nanoparticles and exposed to AMF (+AMF+MNP). Once cells were approximately 85% confluence, APS-SPIONs were added to the culture dishes (−AMF+MNP) and (+AMF+MNP) at different concentrations: 1 mg/mL and 4 mg/mL for 24 h and 2 h of incubation, respectively. Subsequently, the culture dishes (+AMF−MNP) and (+AMF+MNP) were placed with the solenoid coil under the AC external magnetic field set at TS/SN for 2.14 mT/200 kHz, TP/SN for 2.78 mT/300 kHz, and TR/SN for 3.42 mT/300 kHz within an exposition period of 30 min. The treatment time in our study was applied for 30 min to prevent extreme coil overheating and damage to the system. All experiments were initiated at 37 °C, which is the body’s physiological temperature. The frequency and amplitude of the nonharmonic signals (TR, TP, TS) were chosen based on the values that resulted in the highest increase in temperature inside the cell culture medium (1–1.5 °C) in the presence of APS-SPION and, therefore, the same frequency and amplitude parameters were set for their corresponding harmonic signals (SN). In addition, we were unable to create the TP and TS signals at 300 kHz and 3.42 mT due to coil overheating and substantial interferences. Generating pure TP and TS signals at the magnetic field parameters of 300 kHz and 3.42 mT was not possible with our AMF applicator. For this reason, the amplitude for TP needed to be reduced to 2.78 mT while keeping the same frequency. Both frequency and intensity of TS needed to be reduced to 200 kHz and 2.14 mT. In all cases, no increase in temperature was observed during the exposure to only AMF (Figure 3A). The use of low amplitude values will not significantly affect the generation of heat since it has been demonstrated that signals with high slopes could induce higher power losses without the need to use very high magnetic field amplitudes [34].

### 4.6. Post-Magnetic Hyperthermia Calcein-AM/PI Staining

In the next section, we present the procedure employed for cell viability examination after the hyperthermic treatment on our cell lines (B16F10 and CT2A) exposed to TP, TS, and TR and compared to SN waveform using the same frequency and amplitude parameters for 30 min.

Calcein-AM and Propidium Iodide (PI) staining was used to qualitatively measure the cell viability after various hyperthermic treatments applied to the B16F10 and CT2A cell lines. Cell viability was measured by determining the percentage of alive/dead cells relative to the total number of cells in the P35 dish. The cells were incubated for 15–20 min, with the two fluorescent markers Calcein-AM for labeling viable cells and PI for staining the nucleus of dead cells. Observations and image acquisitions were carried out using the DMI 3000B Fluorescence Microscope (Leica, Nussloch, Germany). Calcein-AM is a hydrophobic fluorogenic compound that passively crosses the plasma membrane of cells. Once in the cytoplasm, intracellular esters hydrolyze Calcein-AM to a fluorescent green derivative in viable cells [55]. Propidium iodide is a red fluorescent nuclear marker impermeable to the cellular membrane. Therefore, the nuclei of cells with damaged membranes (necrotic cells) will be stained with this dye [56]. The cells were hence stained with a combined solution of 0.5 μM Calcein acetoxymethyl (Invitrogen, Eugene, OR, USA) and 0.75 μM Propidium Iodide (Sigma-Aldrich, St. Louis, MO, USA) prepared in DMEM for approximately 10–15 min at 37 °C. Cells were imaged after incubation with the imaging system of the Fluorescence Microscope. The excitation light wavelength for Calcein-AM and PI was 495 nm and 535 nm, and the emission light wavelength was 515 nm and 617 nm, respectively. To illustrate the results obtained, the GraphPad Prism and ImageJ programs were implemented. We selected this technique to evaluate the cell viability rather than others like XTT because it allows us to observe modifications in cell morphology. Cell viability of the −AMF−MNP group was measured for the sake of comparison with the other predefined groups (+AMF−MNP, −AMF+MNP, +AMF+MNP), while the cell viability of the +AMF+MNP group, using sinusoidal signal (SN), was evaluated for comparison with the +AMF+MNP group using the nonsinusoidal signals (TP, TS, TR).

### 4.7. Cytotoxicity Studies

The potential cytotoxicity sof the APS-SPION on B16F10 and CT2A cell lines was investigated using the XTT (2,3-bis-(2-methoxy-4-nitro-5-sulfophenyl)-2H-tetrazolium-5-carboxanilide) (AppliChem GmbH, Darmstadt, Germany) colorimetric assay [57] to check the effect of MNPs on both cell lines and to select the adequate APS-SPION concentrations to use during MHT in vitro tests. For the XTT assay, B16F10 and CT2A cells were seeded in 24-well plates at a density of 3 × 10^4^ cells/cm^2^ and 1.6 × 10^4^ cells/cm^2^, respectively, and incubated at 37 °C for 24 h. Next, the cell culture medium was removed, and fresh medium containing 1 and 5 mg Fe/mL of APS-SPION was added for 6 h, 24 h, and 48 h. Untreated cells were used as controls. Later, the medium was discarded, and the wells were washed twice with DMEM and incubated with 350 µL of XTT master mix for an additional 1 h at 37 °C. After that, the absorbance of each well was determined at 470 nm using an ELX808 microplate reader (BioTeK, Winooski, VT, USA) to estimate the cell viability. The absorbance value of the control was equated to 100%, and all the rest of the values were calculated relative to the control. The assay was repeated in triplicate.

Cell viability was calculated according to the following formula:

$$\mathrm{Cell}\;\mathrm{viability}\;\%=\frac{\mathrm{average}\;\mathrm{absorbance}\;\mathrm{from}\;\mathrm{treated}\;\mathrm{cells}}{\mathrm{average}\;\mathrm{absorbance}\;\mathrm{from}\;\mathrm{control}\;\mathrm{cells}}\times 100$$

### 4.8. Statistical Analysis

The obtained data were statistically analyzed using the software package GraphPad Prism Version 8.0.1 for Windows. Data were tested by Shapiro–Wilk test for normality and presented as mean ± SEM (n = 3). The differences between data groups were calculated using the ANOVA test, considered of statistical significance at *p* < 0.05.

### 4.9. Cellular Localization of MNPs

To determine the localization of APS-SPION, an antibody targeted against lysosomes, anti-CD63 antibody (Abcam, Carlsbad, CA, USA), was used. B16F10 and CT2A cells were grown on coverslips at a density of 1.6 × 10^4^ cells/cm^2^ and 3 × 10^4^ cells/cm^2^, respectively, and incubated with 10.6 nm nanoparticles at 1 mg/mL for 24 h. Then, the cells were rinsed 3 times with PBS to remove the excess nanoparticles and fixed using 4% paraformaldehyde (Sigma-Aldrich, St. Louis, MO, USA) in 0.1 M phosphate buffer at pH 7.4 for 30 min at room temperature. The fixed cells were washed 3 times in PBS and blocked for 1 h with a PBS solution containing 10% horse serum (Gibco, Carlsbad, CA, USA) and 0.25% Triton X-100 (Sigma Aldrich, USA). Later, the cells were incubated overnight at 4 °C with an anti-CD63 antibody diluted to 1:500 in PBS solution with 0.25% Triton and 1% horse serum. After removing the solution with the primary antibody and rinsing, samples were incubated with antirabbit Alexa Fluor™ 488 secondary antibody (Abcam, USA) diluted to 1:500 in PBS solution with 0.25% Triton and 1% horse serum for 30 min at room temperature. Finally, after staining, the coverslips were washed with distilled H2O, allowed to dry, and mounted with ProLong (Invitrogen, Eugene, OR, USA).

## 5. Conclusions

Until today, the conventional sinusoidal signal was exceptionally applied in hyperthermic research against cancer. The present study demonstrates the excellent therapeutic efficacy of nonsinusoidal signals TP, TS, and TR for enhancing MHT treatment against cancer cells. The coaction of new nonharmonic signals in the presence of SPIONs can create a substantial improvement for cancer treatment by producing a higher impact inside cells over the harmonic one. The results revealed that magnetic-hyperthermia-mediated-nonsinusoidal waveforms increase tumor cell death compared to the conventional sinusoidal signal. The synergetic effect of AMF and MNPs is responsible for cell death without a perceptible increase in temperature to the range of mild hyperthermia inside the culture medium. In addition, various cancer cell lines have been shown to respond differently to the treatment. The CT2A cell line has shown higher sensitivity to the treatment and, therefore, higher mortality than B16F10. A future study could be oriented toward the investigation of the optimum magnetic field (frequency, amplitude) and the optimum MNP conditions (concentration, incubation period, treatment period) to increase the rate of cell death with nonsinusoidal signals. We have demonstrated the safety of the AMF applicator and the absence of eddy currents or any external interferences that could come from the coil or the surrounding environment and affect the viability of our cells. In parallel, we showed the intracellular localization of APS-SPION in both B16F10 and CT2A cells. The application of TP, TS, or TR waveforms has more effective and considerable consequences on the viability of cancer cells; therefore, the waveform of AMF must be considered as an additional parameter to optimize MHT and not be limited to only the frequency and the amplitude of AMF or the composition and size of the nanoparticles. We conclude that hyperthermia therapies using nonharmonic waveforms offer substantial advantages over conventional harmonic waveforms, especially by using biocompatible superparamagnetic iron oxide nanoparticles of small size that can easily cross biological barriers.

## Data Availability

The presented study data are available on request from the corresponding author.
